# Bigeminy and the bifid papillary muscle

**DOI:** 10.1186/1476-7120-8-13

**Published:** 2010-04-21

**Authors:** James Ker

**Affiliations:** 1 Department of Physiology, University of Pretoria, Pretoria, South Africa; 2 PO Box 24318, Gesina, Pretoria, 0031, South Africa

## Abstract

Various structural anomalies of the left ventricular papillary muscles have been observed in recent years. Many of these have been linked to electrocardiographic aberrations.

Recently two reports have appeared where the base of the posterior papillary muscle was identified as the source of frequent premature ventricular complexes. In some of these patients these frequent premature ventricular complexes have led to left ventricular dysfunction.

In this report a newly discovered structural variant of the anterior papillary muscle is described--the bifid papillary muscle. Furthermore, it is proposed that this bifid papillary muscle is the source of frequent ventricular premature complexes, presenting as bigeminy in a patient with normal left ventricular function.

## Introduction

In recent years various anomalies of the left ventricular papillary muscles have been observed [1]. These include: solitary hypertrophy [2] (as a variant of hypertrophic cardiomyopathy), accessory papillary muscles [1], inverted papillary muscles giving a "mirror" appearance [3], and an octopus-shaped variant, leading to mid-ventricular obstruction [4].

Interestingly, many of these papillary muscle variants have been linked to electrocardiographic aberrations [1,2,5]: These include: prominent U-waves in the inferior leads with an accessory papillary muscle [1], notching of the QRS-complex with ST-segment elevation and a prominent, positive U-wave, all in lead V4 with solitary hypertrophy of the anterolateral papillary muscle [2] and inferior J-waves with an accessory papillary muscle [5].

In this report, a new structural variant of the anterolateral papillary muscle is described--the bifid papillary muscle. In addition the patient had frequent premature ventricular complexes, presenting with pulsus bigeminy. It is proposed that the bifid papillary muscle is a newly discovered entity causing bigeminy.

## Case report

A case report is presented depicting a new variant of the anterolateral papillary muscle--the bifid papillary muscle. Furthermore, there is a growing number of reports in the literature demonstrating various electrocardiographic aberrations caused by the papillary muscles and it's variants. Here it is proposed that the bifid papillary muscle is the cause of frequent premature ventricular complexes, presenting clinically with pulsus bigeminy.

A 51-year old Italian woman was referred for a cardiovascular examination by her primary care physician. The reason for the referral was the presence of an irregular pulse beat detected by the primary care physician. The patient herself was totally asymptomatic and was totally unaware of this irregularity. She never had any medical problems, never underwent any surgical procedures and was not taking any medication or illicit drugs. She also never smoked or has any known allergies. The reason for her visit to the primary care physician was for a routine medical examination to ensure that she was fit for travel and for advice on the prevention of influenza H1N1 as she was planning a boat cruise for vacation purposes.

Clinical examination revealed a pulsus bigeminy. The rest of the clinical examination did not reveal any pathological findings and the blood pressure was 115/75 mmHg. The electrocardiogram demonstrated frequent premature ventricular complexes with bigeminy (see additional file 1). The total number of premature ventricular complexes amounted to 21345 over a 24-hour period. A comprehensive biochemical screen did not reveal any pH, electrolyte or hematological abnormalities which could account for the rhythm disturbance. This biochemical screen specifically excluded thyroid abnormalities. Coronary artery disease was ruled out by means of a CT-angiogram. Although the patient had a low risk for atherosclerotic disease this was done to exclude the possibility of alternative ischaemic etiologies, such as coronary artery anomalies.

Echocardiography demonstrated a peculiar anomaly of the anterolateral papillary muscle (see additional files 2, 3, 4 and 5). The apical, two chamber view revealed a bifid appearance of the anterolateral papillary muscle (see additional files 2 and 3).

The parasternal, short axis view also clearly demonstrates the bifid appearance (see additional files 4 and 5). The larger of the two heads are marked with +. Note the smaller head below. Additional files 6, 7 and 8 demonstrate additional apical two chamber images.

Due to the fact that papillary muscle abnormalities are frequent in patients with hypertrophic cardiomyopathy (HCM), it was specifically noted that there were no features of hypertrophic cardiomyopathy present in this case. There were no ventricular hypertrophy, systolic anterior motion of the mitral valve, septal immobility and the septal to free wall thickness ratio was < 1.3. Specifically there were also no apical hypertrophy or any T wave inversion, suggestive of apical HCM.

## Discussion

Premature ventricular complexes (ventricular premature beats) is the most common arrhythmia detected by physicians during a physical examination [6].

Recently it was realized that the papillary muscles of the left ventricle may be the source of frequent premature ventricular complexes [7,8].

Doppalapudi et al [7] recently described a distinct new syndrome in seven patients. In this syndrome ventricular arrhythmia arises from the base of the posterior papillary muscle in the left ventricle. This entity presented as sustained ventricular tachycardia in two patients and as frequent premature ventricular complexes with salvos of non-sustained ventricular tachycardia in five patients [7]. Importantly, all these patients had a normal left ventricular function.

In contrast to this series with normal left ventricular function Sternick et al [8] published a case where frequent premature ventricular complexes, also arising from the base of the left posterior papillary muscle provoked significant left ventricular dysfunction. Furthermore, after cool-tip ablation with resultant elimination of these premature ventricular complexes, complete reversal of the left ventricular dysfunction occurred.

In conclusion, a case is presented of a newly described structural variant of the left anterolateral papillary muscle--the bifid papillary muscle. It is furthermore also proposed that this structural variant is the source of frequent premature ventricular complexes, presenting as bigeminy in a patient with normal left ventricular function. This adds to the cited two reports where the left ventricular posterior papillary muscle was the source of frequent premature ventricular complexes.

## Competing interests

The author declares that they have no competing interests.

## Authors' contributions

JK is the sole author.

## Supplementary Material

Additional file 1 **Electrocardiogram demonstrating frequent premature ventricular complexes with bigeminy**. This is the 12 lead electrocardiogram, demonstrating frequent premature ventricular complexes with bigeminy. Click here for file

Additional file 2 **The bifid papillary muscle**. This is an image from the apical two chamber view, clearly demonstrating the bifid anterolateral papillary muscle. Click here for file

Additional file 3 **Apical two chamber view**. This is a movie clip from the apical two chamber view, demonstrating the bifid anterolateral papillary muscle. Click here for file

Additional file 4 **Parasternal short axis view**. This is an image from the parasternal short axis view, clearly demonstrating the bifid anterolateral papillary muscle. The larger upper head is marked with +. Note the smaller head below. Click here for file

Additional file 5 **Parasternal short axis view**. This is a movie clip from the parasternal short axis view, demonstrating the bifid anterolateral papillary muscle. Click here for file

Additional file 6 **Apical two chamber view**. This is an additional movie clip from the apical two chamber view. Click here for file

Additional file 7 **Apical two chamber view**. This is an additional movie clip from the apical two chamber view. Click here for file

Additional file 8 **Apical two chamber view**. This is an additional movie clip from the apical two chamber view. Click here for file
