# Supplementary figures and images for: Bigeminy and the bifid papillary muscle
Source: Cardiovasc Ultrasound. 2010 Apr 21;8:13. doi: 10.1186/1476-7120-8-13 (PMC2867800; doi:10.1186/1476-7120-8-13)

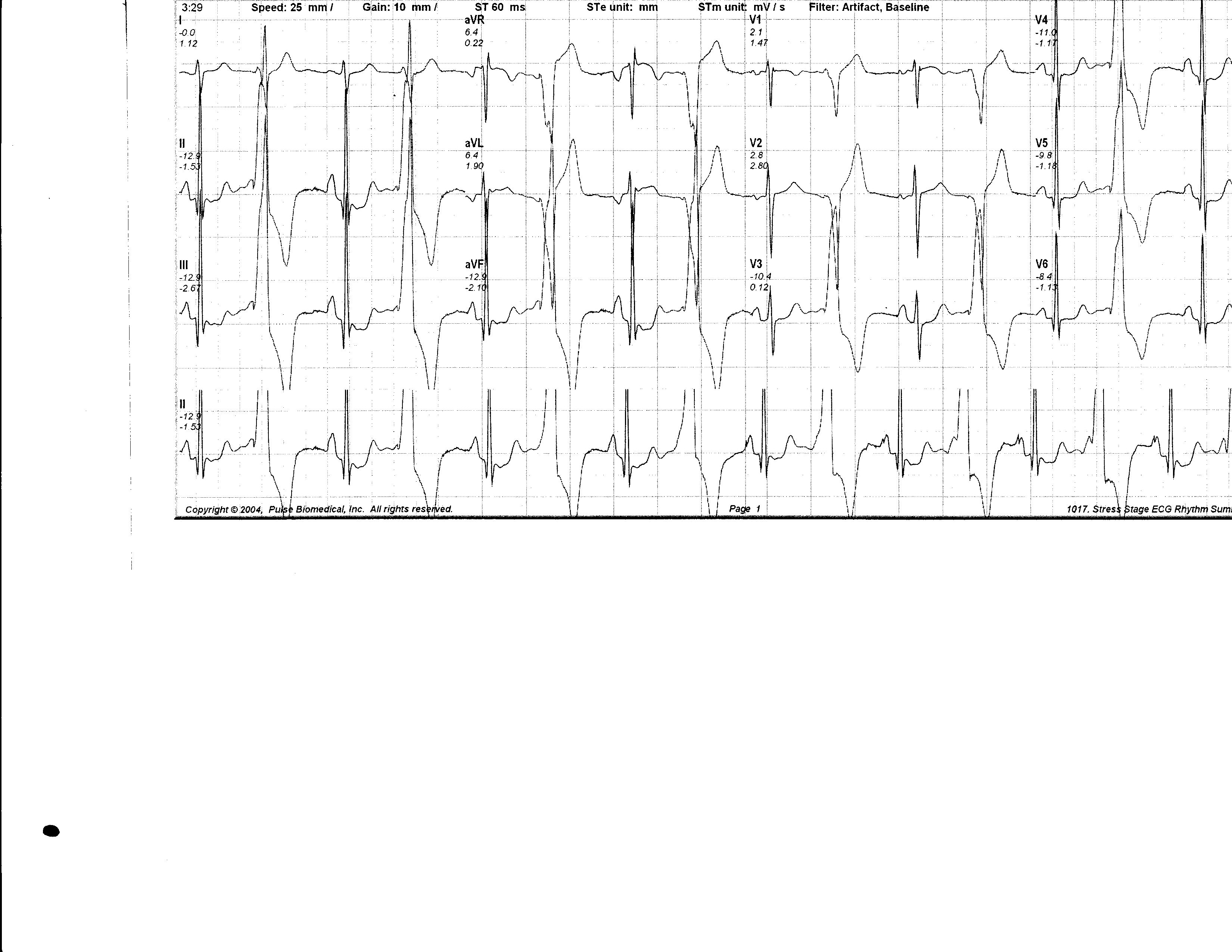

Supplement: Additional file 1 — Electrocardiogram demonstrating frequent premature ventricular complexes with bigeminy. This is the 12 lead electrocardiogram, demonstrating frequent premature ventricular complexes with bigeminy. [file 1476-7120-8-13-S1.JPEG]

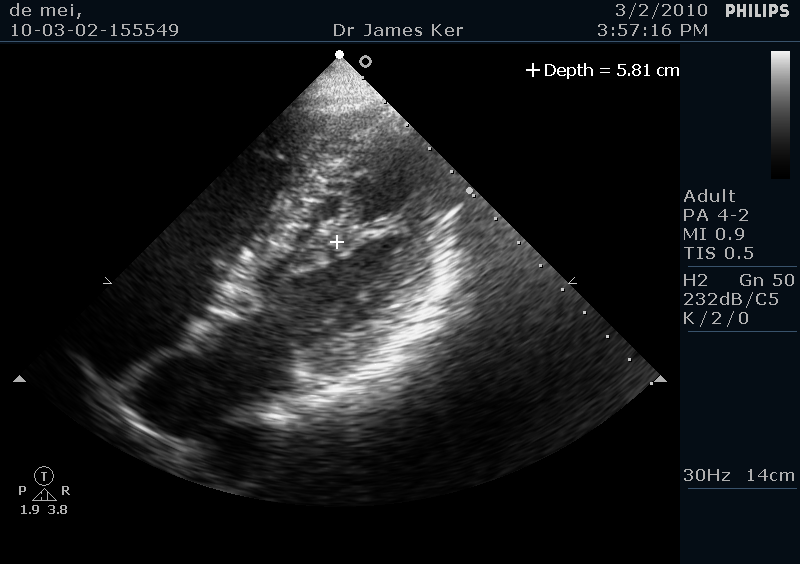

Supplement: Additional file 2 — The bifid papillary muscle. This is an image from the apical two chamber view, clearly demonstrating the bifid anterolateral papillary muscle. [file 1476-7120-8-13-S2.BMP]

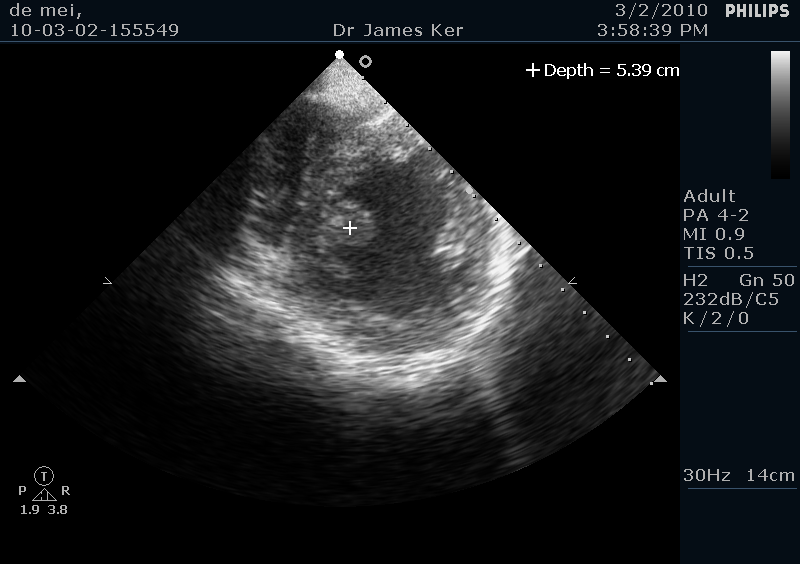

Supplement: Additional file 4 — Parasternal short axis view. This is an image from the parasternal short axis view, clearly demonstrating the bifid anterolateral papillary muscle. The larger upper head is marked with +. Note the smaller head below. [file 1476-7120-8-13-S4.BMP]
